# Eliminating Cervical Cancer in India: Challenges and Prospects

**DOI:** 10.4269/ajtmh.25-0396

**Published:** 2025-11-11

**Authors:** Sanjanaa Senthilkumar, Rohit A. Chitale, Prakasha Kempaiah, Deepika Saraf, Ravi Durvasula

**Affiliations:** ^1^ Department of Infectious Diseases, Mayo Clinic, Jacksonville, Florida;; ^2^ Centre for Health Innovation and Policy, Noida, India

## Abstract

Cervical cancer is a malignancy with a well-established viral origin linked to high-risk human papillomavirus (HPV) types. Globally, there are 348,709 estimated deaths among 662,301 cervical cancer cases. India faces a disproportionately high burden, contributing to 79,906 of these deaths and accounting for over 22% of global deaths. The nation’s high mortality rate is attributable to the low rate of cervical cancer screening and HPV vaccination uptake. There is a greater impact of cervical cancer on those of lower socioeconomic status and a cultural stigma surrounding sexually transmitted infections. We recommend a comprehensive cervical cancer prevention strategy, including screening programs, culturally sensitive education for patients and providers, government-endorsed HPV vaccination, and continuing studies on diagnostics and vaccination in the Indian context.

## INTRODUCTION

### The persistent crisis of cervical cancer in India.

Cervical cancer remains an urgent global health challenge that accounts for significant morbidity and mortality. Globally, in 2022, an estimated 662,301 women were newly diagnosed with the disease, with 348,709 deaths. In India, 123,907 women were diagnosed with cervical cancer in 2022.^1^^,^^2^ India faces a disproportionately high burden, accounting for over 23% of global deaths caused by cervical cancer.^3^ The nation’s alarming cervical cancer mortality rate is attributable to the low rate of cervical cancer screening (2%) and resistance to human papilloma virus (HPV) vaccination.^3^ Although India’s screening rate remains low, other low- to middle-income countries (LMICs) have achieved higher screening rates (e.g., Kenya at 16.4%, Vietnam at 25%, and Iran at 52.1%), highlighting the potential for improvement through targeted public health initiatives.^4^ Epidemiological data expose major gaps in vaccination enrollment, cervical cancer screening, and sexual education among school-aged girls.^5^^,^^6^ Cervical cancer disproportionately affects women from low socioeconomic strata because of the unaffordability of vaccines, lack of early detection through screening, and other access barriers to treatments.^7^^,^^8^ The barriers are not limited to economics; there is significant cultural stigma surrounding sexually transmitted infection (STI) rooted in moral judgment and generational misconceptions.^9^ The taboo of STIs must be addressed, without which low screening and vaccination uptake will persist, ultimately hindering care for millions of women.

### Educational, cultural, economic, and political factors hindering cervical cancer prevention in India.

Most cervical cancers start as HPV infections, which have potential to progress into malignancy.^10^ The current HPV vaccines target high-risk HPV types. Regular Pap smear tests and HPV DNA testing help detect precancerous changes and enable early intervention. This pathophysiology underscores the importance of vaccination, regular screening, and early treatment to reduce the risk of developing cervical cancer. More specific to India, risk factors for cervical cancer include early marriage, multiple pregnancies, malnutrition, poor genital hygiene, and having a spouse who has had multiple sexual partners.

The paucity of education in India on this issue has unfortunate and tangible repercussions. The low awareness of HPV’s link to cervical cancer causes increased vaccination reluctance. A study done in the state of Madhya Pradesh with students ages 11–16 years old revealed that only about 34% of female students had heard of cervical cancer and that only 7.8% of female students were willing to take the vaccine. Students showed optimism toward the idea of future cervical cancer awareness and outreach programs at schools. Among those who already knew about cervical cancer, only 17.9% attributed the education to health care professionals.^11^ This aligns with research that demonstrates high physician hesitancy when it comes to patient communication about the HPV vaccine.^12^ Another study conducted on female health care students found that students missed key details about HPV transmission, prevention, and vaccination.^13^

India’s approach to sexual education, especially for female sexual health, has always been a cause for distress at the government and community levels.^14^ Adolescents in India are unable to receive proper sexual education because public discussion of sexually suggestive subjects is generally seen as taboo in Indian society.^15^ Unfortunately, STIs, contraceptive use, and preventive measures such as Pap smears are rarely incorporated into this education.^16^ Thus, implementing comprehensive sexual education in the Indian population is very challenging.^17^

Unfortunately, suboptimal uptake is observed not only in cervical cancer screening but also, in breast cancer screening. The prevalence of mammography among Indian women ages 45 years old and older has been reported to be 1.3%, with rates of 1.7% among those ages 45–59 years old and 0.9% among women ages 60 years old and older.^18^ These low screening rates underscore the limited uptake of these services by the population.

Several studies indicate that women presenting with cervical cancer are often in the end stage, highlighting the lack of accessible preventive and screening measures. Direct costs of the disease, including the costs of surgery, radiotherapy, and chemotherapy, range from 526 to 1,461 U.S. dollars (USD; 125,000 Indian Rupee (INR)) per patient.^8^ Indirect costs include losses in productivity and loss of quality of life. In 2016, India recorded a burden of cervical cancer of 223.8 disability-adjusted life years (DALYs) per 100,000 women.^19^ The DALYs were the highest in the northeast region (290.1 DALYs per 100,000 women). Projections for 2025 are slated to reach 1.5 million DALYs.^19^

India has taken strides toward addressing cervical cancer prevention, but the HPV vaccine has not yet been officially incorporated into the Universal Immunization Program (UIP), attributed largely to the lack of funding and shared sense of urgency.^20^ The National Technical Advisory Group on Immunization (NTAGI) has already suggested adding the vaccine to the UIP. International groups, like Gavi, the Vaccine Alliance, and WHO, help with vaccine projects in LMICs, including India. The Union for International Cancer Control started grants to increase civil society organizations’ impact on HPV vaccination access, matching efforts to eliminate cervical cancer globally. The Indian Medical Association, the Federation of Obstetric and Gynaecological Societies of India, and the Indian Academy of Pediatrics Committee on Immunization are just a few of the organizations within India that aim to increase physician and community awareness on the HPV vaccine and cervical cancer. Without government endorsement, however, this task is too immense to percolate down to the beneficiaries. From a societal acceptance perspective, individuals will be more likely to trust the vaccine once the government endorses it in the UIP.

Gardasil, a quadrivalent vaccine, and Cervarix, a bivalent vaccine, are the two globally licensed HPV vaccines that were initially available in India. The price of vaccination was high, with two doses each at around 48 USD (4,000 INR). Cervavac, India’s first local HPV vaccine manufactured by the Serum Institute of India, brought this cost down to 24 USD (2,000 INR) per dose—still unaffordable for many people.^20^ In January 2023, the Indian Ministry of Health and Family Welfare wrote to several state governments to prepare for vaccine rollout later that year. Per the plan, other states and territories were to follow this rollout in 2024 and 2025.^21^ Given that Cervavac was ready to be administered in 2023, this message seemed promising. In February 2024, the Indian Finance Minister announced that there would be government aid to make vaccines more affordable, and there was a movement to include the HPV vaccine in the UIP “in the near future.” For at least a decade, the government has intended to implement large state-level interventions to reduce the burden of cervical cancer, but thus far, it has not done so.

### India’s successful vaccination programs.

India’s past vaccination successes are encouraging. In January 2021, India’s coronavirus disease 2019 (COVID-19) vaccination program was launched, and by January 2023, it had administered 2.2 billion doses, covering 97% of eligible citizens with at least one dose. The implementation efficiency of administrating vaccines to populations in rural settings demonstrates India’s capabilities and resources. Polio elimination in India is another excellent example of how the country mobilized a vaccine against a crippling infectious disease. The polio vaccine was one of the first vaccines included in the UIP, and its success was a result of meticulous strategic execution. The Pulse Polio Immunization Program ensured that every child younger than 5 years of age was vaccinated.^22^ India achieved polio-free status in 2014 in large part because of its vaccination policy. The Government of India’s support for the prevention and control of hepatitis B (HepB) provides a compelling example of how a vaccine can prevent cancer, specifically hepatocellular carcinoma (HCC). As of 2015, the three-dose HepB vaccination rate was about 84% in infants.^23^ This led to significantly reduced HepB-associated hepatitis and will likely lead to decreased HCC as seen in other nations.^24^^,^^25^

### Strategic approaches to prevent cervical cancer in India.

Given the underlying drivers leading to the persistence of cervical cancer, we propose a three-pronged comprehensive cervical cancer prevention strategy.

#### Integrating the HPV vaccine into government policy and community.

Inclusion of the HPV vaccine into UIP is a pivotal next step that can dramatically increase uptake of HPV among young girls. Global evidence shows that prophylactic vaccination significantly lowers the risk of high-risk HPV infection.^26^ Not only will the vaccine become more financially accessible, but it will also send a powerful political and public health message, demonstrating that cervical cancer prevention is a national priority.

Although policy plays a crucial role in breaking down logistical barriers, community-based outreach is key to prevent misinformation and address vaccine hesitation. Targeted campaigns must proactively engage with adolescents, parents, teachers, and health care workers. The organizations should focus on strategically delivering the large-scale intent of the vaccine, emphasizing that this is a national issue. The message must be culturally sensitive and tailored to school-based or community-based events.

#### Rethinking HPV prevention for women in India.

In India, girls and women are more likely to care for sick family members and often assist during hospitalizations.^27^^,^^28^ This gender-biased cultural norm should be leveraged as a point of interaction between women and large-scale health infrastructure. For example, at large government hospitals, a unit could focus on vaccinating girls and women, even if they were not present for their own care. Given that women in India are less likely to seek medical care for themselves, every interaction with the health care system should be used as a potential opportunity for vaccination and cancer prevention.^29^^,^^30^ This vaccine-recruiting method can also be implemented to new mothers younger than 26 years of age at follow-up visits or during postpartum hospitalization. The American College of Obstetrics and Gynecology emphasizes that the HPV vaccine may be given any time after birth and is safe during breastfeeding. Evidence on the efficacy of the single-dose HPV vaccine is promising and further mitigates the need for multiple visits to the clinic, thereby increasing adherence.^31^

A Google trends study provides encouraging results that show increased interest in the topic of HPV vaccines. Searches spiked in 2018 when the NTAGI recommended the vaccine and again in 2022 when India launched its own HPV vaccine, indicating a growing public interest and underscoring the need for government and public health departments to prioritize access and delivery of the HPV vaccine.^32^ At-home HPV testing should also be strongly considered. Although such tests are gaining traction in developed countries, similar studies should be pursued in India.^33^^,^^34^ At-home testing helps mitigate discomfort and stigma surrounding pelvic examinations and Pap smears. These should be made available at schools, universities, and clinics. There should be clear instructions on next steps in case of positive results.

#### Navigating cervical cancer in India.

Identifying early cervical lesions is extremely challenging given that cervical cancer screening with pelvic examination occurs in less than 2% of women in India.^35^ It is thereby critical to emphasize symptoms that should not be overlooked. Unfortunately, an individual with early-stage cervical cancer may be asymptomatic. As malignancy progresses, symptoms, such as abnormal vaginal bleeding, pelvic pain, and vaginal discharge, may present. Campaigns that promote HPV prevention should also educate the public about symptoms of cervical cancer. More importantly, it is important for individuals to seek a provider when they notice these symptoms. Health care providers should also pay attention to urogenital symptoms and have a lower threshold to test for cervical cancer. Despite prevailing social norms, sexual history taking is strongly advised to best assess cervical cancer risk. The limited access to radiotherapy/brachytherapy for higher-grade lesions makes it difficult for individuals to receive adequate treatment in LMICs.^36^ Furthermore, the financial barriers are significant, often discouraging women away from seeking care. Government must make strides to expand access to affordable treatment services, including appropriate infrastructure for cancer care. State governments should subsidize transportation costs for women referred for cervical cancer screening or treatment, especially in rural blocks, inspired by existing schemes, like Janani Shishu Suraksha Karyakram.^37^ Furthermore, public–private partnerships should be created to establish at least one radiotherapy facility per district, with fast-tracked referral service for those who have higher-stage lesions.

## CONCLUSION

Cervical cancer remains a significant yet preventable public health issue in India that is exacerbated by a confluence of cultural, educational, socioeconomic, and political factors. Despite clear scientific evidence of the effectiveness of HPV vaccination and early screening methods, the implementation of widespread prevention strategies remains insufficient. The burden disproportionately affects women from underserved communities, where access to health care, education, and accurate information is limited. Cultural stigmas surrounding STIs further hinder awareness, communication, and vaccine uptake. Current efforts by local and international health organizations highlight progress, but without strong government commitment, particularly in incorporating HPV vaccination into UIP, these interventions will remain fragmented and inadequate. India has proven its capacity to execute large-scale vaccination campaigns as seen with the successful rollouts of the polio and COVID-19 vaccines. Drawing from these models, a comprehensive cervical cancer prevention strategy must include government-endorsed HPV vaccination, accessible screening programs, and culturally sensitive education initiatives. Bridging the knowledge gap through school-based health education, empowering health care workers to communicate effectively, and engaging families can combat stigma and drive community participation. With strategic investment and unified effort, India could significantly reduce the incidence and mortality of cervical cancer, saving lives and advancing equity in women’s health.

## References

[1] Krishnan S Madsen E Porterfield D Varghese B, 2013. Advancing cervical cancer prevention in India: Implementation science priorities. Oncologist 18: 1285–1297. 24217555 10.1634/theoncologist.2013-0292 PMC3868423

[2] Rai R, , 2023. Cervical cancer screening coverage at tertiary care institutes across India. Asian Pac J Cancer Prev 24: 4269–4275. 38156863 10.31557/APJCP.2023.24.12.4269 PMC10909083

[3] Muthuramalingam MR Muraleedharan VR, 2023. Patterns in the prevalence and wealth-based inequality of cervical cancer screening in India. BMC Womens Health 23: 337. 37365552 10.1186/s12905-023-02504-y PMC10291770

[4] Wu J, , 2025. Global burden of cervical cancer: Current estimates, temporal trend and future projections based on the GLOBOCAN 2022. *J Natl Cancer Cent* 5: 322–329. 40693230 10.1016/j.jncc.2024.11.006 PMC12276544

[5] Alekhya G Chinnadurai A Dora S Patro SK Sahu DP Mourougan M, 2025. “Sexuality education is a double edge-sword …”: A qualitative study on perceptions of school teachers on sexual and reproductive health of adolescent girls in Eastern India. Reprod Health 22: 145. 40790752 10.1186/s12978-025-02098-8 PMC12341063

[6] Sundriyal D Bahurupi Y Rajaram S Singh M Aggarwal P Antil P Gupta S Sehrawat A, 2025. *Perspective of Females from a Rural Community in India toward the Knowledge, Screening, and Treatment of Breast and Cervical Cancer and Preventive Vaccination*. Available at: 10.1007/s10552-025-02018-y. Accessed October 22, 2025. 40504335

[7] Gupta S Dey A Kundu S Sinha Gupta S, 2025. Barriers to cervical cancer screening in India: Insights from National Family Health Survey-5 data. Asian Pac J Cancer Prev 26: 1853–1861. 40439399 10.31557/APJCP.2025.26.5.1853 PMC12290191

[8] Singh MP Chauhan AS Rai B Ghoshal S Prinja S, 2020. Cost of treatment for cervical cancer in India. Asian Pac J Cancer Prev 21: 2639–2646. 32986363 10.31557/APJCP.2020.21.9.2639 PMC7779435

[9] Poddar A Rao SR, 2024. Overcoming barriers of cervical cancer elimination in India: A practice to policy level advocacy. J Cancer Policy 43: 100521. 39542420 10.1016/j.jcpo.2024.100521

[10] Burd EM, 2003. Human papillomavirus and cervical cancer. Clin Microbiol Rev 16: 1–17. 12525422 10.1128/CMR.16.1.1-17.2003 PMC145302

[11] Dewan M Shukla D Shrivastav G Diwan P, 2024. Knowledge and attitude towards HPV infection, HPV vaccination and cervical cancer among middle- and high-school students in Gwalior. J Family Med Prim Care 13: 4382–4387. 39629380 10.4103/jfmpc.jfmpc_389_24 PMC11610844

[12] Kataria I, , 2022. Awareness, perceptions, and choices of physicians pertaining to human papillomavirus (HPV) vaccination in India: A formative research study. Vaccine X 12: 100228. 36317080 10.1016/j.jvacx.2022.100228 PMC9617192

[13] Verma I Bajpai R Arjaria V Garg L Mungad A Singh D Gavli J Khare A, 2024. A study to assess the impact of education on the knowledge and attitude toward cervical cancer and HPV (human papillomavirus) vaccination among female healthcare students. Cureus 16: e59856. 38854271 10.7759/cureus.59856 PMC11157296

[14] Khubchandani J Clark J Kumar R, 2014. Beyond controversies: Sexuality education for adolescents in India. J Family Med Prim Care 3: 175–179. 25374847 10.4103/2249-4863.141588 PMC4209665

[15] Ismail S Shajahan A Sathyanarayana Rao TS Wylie K, 2015. Adolescent sex education in India: Current perspectives. Indian J Psychiatry 57: 333–337. 26816418 10.4103/0019-5545.171843 PMC4711229

[16] Pattathil N Roy A, 2023. Promising practices for the design and implementation of sexuality education programmes for youth in India: A scoping review. Sex Reprod Health Matters 31: 2244268. 37712401 10.1080/26410397.2023.2244268 PMC10506433

[17] Joseph JT, 2023. Comprehensive sexuality education in the Indian context: Challenges and opportunities. Indian J Psychol Med 45: 292–296. 37152376 10.1177/02537176221139566 PMC10159571

[18] Sharma P Das D Khanna D Budukh A Khokhar A Pradhan S Khanna AK Chaturvedi P Badwe R, 2025. Prevalence and associated factors of mammography uptake among the women aged 45 years and above: Policy implications from the longitudinal ageing study in India Wave I survey. BMC Public Health 25: 1073. 40108533 10.1186/s12889-025-22261-x PMC11924914

[19] Ramamoorthy T Kulothungan V Sathishkumar K Tomy N Mohan R Balan S Mathur P, 2024. Burden of cervical cancer in India: Estimates of years of life lost, years lived with disability and disability adjusted life years at national and subnational levels using the National Cancer Registry Programme data. Reprod Health 21: 111. 39075548 10.1186/s12978-024-01837-7 PMC11287936

[20] Ahuja M Sharma P Sarkar A, 2022. Universal cervical cancer immunization: India ready for a quantum leap. J Midlife Health 13: 260–262. 36950205 10.4103/jmh.jmh_224_22 PMC10025827

[21] Burki TK, 2023. India rolls out HPV vaccination. Lancet Oncol 24: e147. 36934729 10.1016/S1470-2045(23)00118-3

[22] John TJ Vashishtha VM, 2013. Eradicating poliomyelitis: India’s journey from hyperendemic to polio-free status. Indian J Med Res 137: 881–894. 23760372 PMC3734678

[23] Sandhu HS Roesel S Sharifuzzaman M Chunsuttiwat S Tohme RA, 2020. Progress toward hepatitis B control—South-east Asia region, 2016–2019. MMWR Morb Mortal Wkly Rep 69: 988–992. 32730237 10.15585/mmwr.mm6930a2 PMC7392392

[24] Murhekar MV, , 2020. Hepatitis-B virus infection in India: Findings from a nationally representative serosurvey, 2017–18. Int J Infect Dis 100: 455–460. 32896662 10.1016/j.ijid.2020.08.084

[25] Cao M, , 2022. Long term outcome of prevention of liver cancer by hepatitis B vaccine: Results from an RCT with 37 years. Cancer Lett 536: 215652. 35318115 10.1016/j.canlet.2022.215652

[26] Arbyn M Xu L Simoens C Martin-Hirsch PP, 2018. Prophylactic vaccination against human papillomaviruses to prevent cervical cancer and its precursors. Cochrane Database Syst Rev 5: CD009069. 29740819 10.1002/14651858.CD009069.pub3 PMC6494566

[27] Buckshee K, 1997. Impact of roles of women on health in India. Int J Gynaecol Obstet 58: 35–42. 9253664 10.1016/s0020-7292(97)02887-7

[28] Govil D Sahoo H Chowdhury B James KS, 2024. A qualitative perspective of working women care providers and care receivers on eldercare: A study from India. BMC Geriatr 24: 345. 38627618 10.1186/s12877-024-04782-z PMC11021006

[29] Kapoor M Agrawal D Ravi S Roy A Subramanian SV Guleria R, 2019. Missing female patients: An observational analysis of sex ratio among outpatients in a referral tertiary care public hospital in India. BMJ Open 9: e026850. 10.1136/bmjopen-2018-026850 PMC6687005 31391189

[30] Saikia N Null M Bora JK, 2016. Gender difference in health-care expenditure: Evidence from India Human Development Survey. PLoS One 11: e0158332. 27391322 10.1371/journal.pone.0158332 PMC4938214

[31] Jeong M Jang I, 2025. Comparative effectiveness and immunogenicity of single-dose and multi-dose human papillomavirus vaccination: A systematic review. BMC Public Health 25: 2330. 40610947 10.1186/s12889-025-23496-4 PMC12225068

[32] Mehra R, , 2025. Google trends for the human papillomavirus vaccine in India from 2010 to 2024: Infodemiological study. J Med Internet Res 27: e69729. 40424583 10.2196/69729 PMC12133069

[33] Polman NJ, , 2019. Performance of human papillomavirus testing on self-collected versus clinician-collected samples for the detection of cervical intraepithelial neoplasia of grade 2 or worse: A randomised, paired screen-positive, non-inferiority trial. Lancet Oncol 20: 229–238. 30658933 10.1016/S1470-2045(18)30763-0

[34] Chou HH, , 2024. Consistency in human papillomavirus type detection between self-collected vaginal specimens and physician-sampled cervical specimens. J Med Virol 96: e29426. 38420851 10.1002/jmv.29426

[35] Srivastava AN Misra JS Srivastava S Das BC Gupta S, 2018. Cervical cancer screening in rural India: Status & current concepts. Indian J Med Res 148: 687–696. 30778002 10.4103/ijmr.IJMR_5_17 PMC6396551

[36] Chargari C, , 2022. Increasing global accessibility to high-level treatments for cervical cancers. Gynecol Oncol 164: 231–241. 34716024 10.1016/j.ygyno.2021.10.073 PMC9496636

[37] Janani Shishu Suraksha Karyakaram (JSSK), 2025. *National Health Mission*. Available at: https://nhm.gov.in/index4.php?lang=1&level=0&linkid=150&lid=171. Accessed August 24, 2025.

